# The Pathological Mechanisms and Treatments of Tinnitus

**DOI:** 10.15190/d.2021.16

**Published:** 2021-09-30

**Authors:** Sana Saeed, Qudsia Umaira Khan

**Affiliations:** ^1^CMH Lahore Medical College & Institute of Dentistry, Lahore, Pakistan

**Keywords:** Tinnitus, central gain control theory, thalamocortical dysrhythmia, global tinnitus network, tinnitus pathology, tinnitus treatments.

## Abstract

Tinnitus is defined as the ringing, hissing, clicking or roaring sounds an individual consciously perceives in the absence of an external auditory stimulus. Currently, the literature on the mechanism of tinnitus pathology is multifaceted, ranging from tinnitus generation at the cellular level to its perception at the system level. Cellular level mechanisms include increased neuronal synchrony, neurotransmission changes and maladaptive plasticity. At the system level, the role of auditory structures, non-auditory structures, changes in the functional connectivities in higher regions and tinnitus networks have been investigated. The exploration of all these mechanisms creates a holistic view on understanding the changes the pathophysiology of tinnitus undertakes. Although tinnitus percept may start at the level of cochlear nerve deafferentation, the neuronal changes in the central auditory system to the neuronal and connectivity changes in non-auditory regions, such as the limbic system, become cardinal in chronic tinnitus generation. At the present moment, some tinnitus generation mechanisms are well established (e.g., increased neuronal synchrony) whereas other mechanisms have gained more traction recently (e.g., tinnitus networks, tinnitus-distress networks) and therefore, require additional investigation to solidify their role in tinnitus pathology. The treatments and therapeutics designed for tinnitus are numerous, with varied levels of success. They are generally two-fold: some treatments focus on tinnitus cessation (including cochlear implants, deep brain stimulation, transcranial direct current stimulation and transcranial magnetic stimulation) whereas the other set focuses on tinnitus reduction or masking (including hearing aids, sound therapy, cognitive behavioral therapy, tinnitus retraining therapy, and tailor made notched musical training).  Tinnitus management has focused on implementing tinnitus masking/reducing therapies more than tinnitus cessation, since cessation treatments are still lacking in streamlined treatment protocols and long-term sustainability and efficacy of the treatment. This review will focus on concisely exploring the current and most relevant tinnitus pathophysiology mechanisms, treatments and therapeutics.

## SUMMARY

*1. Introduction*

*2. Mechanism of tinnitus generation*

- * 2.1 Cellular level*

- * 2.2 System level *

- * 2.3 Others *

*3. Treatments and Therapeutics*

- * 3.1 Tinnitus masking/reduction *

- * 3.2 Tinnitus cessation *

*4. Conclusion*

## 1. Introduction

Tinnitus is defined as the ringing, hissing, clicking or roaring sound in the ears (either bilateral or unilateral)^1^. The individual consciously perceives a sound in the absence of any external auditory stimulus^2^. Different classification systems have been developed characterizing tinnitus as pulsatile, subjective or objective, primary or secondary, and acute or chronic. Pulsatile tinnitus is almost always specific to causes that are vascular in origin^3^. Objective tinnitus is heard by the patient and the examiner whereas subjective tinnitus is only heard by the individual^2^. Primary and secondary are on the basis of cause whereas acute and chronic indicate duration for which the patient has experienced tinnitus. Currently, tinnitus is viewed as a symptom of an underlying disease rather than a disease of its own. Therefore, multiple causes have been implicated in tinnitus including but not limited to the following: Meniere's disease, otosclerosis, otitis media, and ototoxic medications^2^. One of the most common risk factors for tinnitus is noise induced hearing loss^4,5^. However, cases exist in which tinnitus can occur as an idiopathic symptom^6^.

Once tinnitus develops, it is likely to be permanent. As a result, for some individuals, tinnitus can become a crippling condition to live with, making their day-to-day tasks difficult to complete. For others, they are able to adapt to this symptom and easily integrate into their normal lives. Regardless, its potentially debilitating nature highlights the importance of understanding tinnitus outside of the condition that caused it.

Despite advancements in the causes of tinnitus and types of tinnitus that exist, there is still contention over its pathophysiological mechanism and consequently, the treatments leading to tinnitus cessation. The pathophysiology of tinnitus is characterized by different aspects. Research done at the cellular level demonstrates tinnitus as an increase in neuronal synchrony (i.e., increased firing rate of neurons simultaneously)^7^. This mechanism, by far, is one of the most studied mechanisms for tinnitus pathology. Previously, the focus was placed on auditory structures such as cochlear nerve deafferentation being the cause of tinnitus^8^. However, tinnitus research has ventured into studying the role of non-auditory structures and tinnitus networks in the brain^8,9^. Evidently, tinnitus pathology research has undergone fundamental changes and continues to do so.

This review aims to thematically provide the current pathophysiological understanding of tinnitus and to analyze the current therapies.

For this review, the following exclusion criteria were implemented: tinnitus in children, tinnitus pathophysiology articles before 2010, tinnitus treatment/therapeutics articles before 2015. The inclusion criteria included articles testing treatments on people or animals.

## 2. Mechanisms of Tinnitus Generation

Previously, tinnitus research focused on the internal ear, cochlear nerves and the auditory system in the pursuit of understanding tinnitus pathophysiology. In more recent years, the focus has shifted to tinnitus networks and their interrelationship with other regions in the brain. The research for what started at the level of the ear has effectively been shifted to understanding it at higher levels in the nervous system.

**Figure 1:**
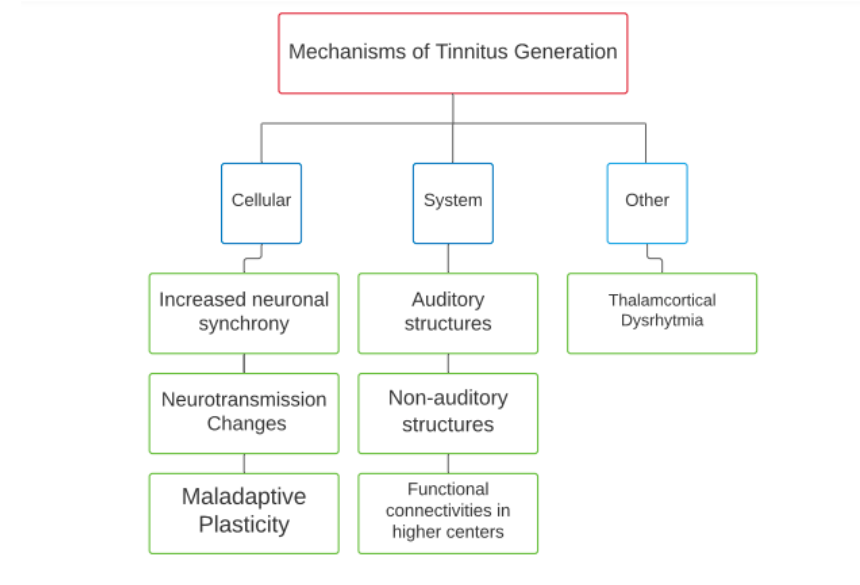
Overview of tinnitus generation mechanisms

The pathologies section thematically covers the different theories and conjectures regarding tinnitus pathology. The mechanisms are discussed under three categories: cellular level, system level and others (Figure 1).

### 2.1 Cellular level

#### 2.1.1 Increase in neural synchrony

The most extensively studied mechanism of tinnitus pathology at the cellular level is increased neuronal synchrony.

The general mechanism starts with noise-induced hearing loss leading to reduced neural input to the central auditory system. As a result, there is increased spontaneous firing rate in the *central auditory system* despite the absence of a physical auditory stimulus^3,8,10-13^. There are slight discrepancies in the location of hyperactivity since studies on the dorsal cochlear nucleus show an increase in neuronal synchrony evidenced by increased spontaneous firing rates (SFRs) in the fusiform cells of the dorsal cochlear nucleus (DCN)^8,10^. Essentially, the neurons are firing action potentials in a synchronized fashion leading to the overall increase in neuronal activity. Animal studies on the medial geniculate body (MGB) demonstrated increased neuronal firing in animals with tinnitus than those without it^8,10^. Therefore, increased neuronal synchrony has an established role in tinnitus pathology and several auditory structures have been implicated in this mechanism. In later sections, the involvement of other auditory and non-auditory structures will also be discussed in the context of this hyperactivity.

This neuronal hyperactivity is also addressed under the “central gain control” theory which states that increased neuronal synchrony occurs in the central auditory system due to the auditory (sensory) deprivation from the cochlea^11^. Again, the mechanism by which hyperactivity occurs is not well understood (can be a decrease in inhibitory synaptic response, increased excitatory synaptics response or altered intrinsic neuronal excitability)^11^.

#### 2.1.2 Neurotransmission changes

Another tinnitus generation mechanism at the cellular level includes changes in neurotransmission. It is important to note that these neurotransmission changes are intrinsically linked to increased neural synchrony which was discussed above. The basic mechanism of cause includes the loss of inhibitory drive which leads to unopposed action of the excitatory drive producing hyperexcitability that is perceived as tinnitus^14^. A study performed by Pilati and colleagues demonstrated a downregulation of high voltage-activated K+ channels in the DCN after intense sound exposure which ultimately resulted in increased incidence of burst responses (i.e., firing rates of neurons)^15^. Another study demonstrated a decrease in presynaptic glutamic acid decarboxylase (GAD) levels in high frequency regions of the primary auditory cortex with a concomitant elevation in firing rates^16^.

Clinical studies testing for GABAA_R_ selective drugs have shown evidence of the link between neurotransmission change and tinnitus. A study on oral dosing of taurine (GABAA_R_ agonist) led to tinnitus attenuation, highlighting how taurine increases inhibition of MGB neurons specifically to decrease the hyperexcitability and thus reduce tinnitus^17^. Another study administered NO-711 and vigabatrin which induced increased GABA levels and again showed similar outcomes to the study above^14^. Furthermore, GABAergic inhibition in the central nucleus of the inferior colliculus (IC) decreased after noise exposure when measured 30 days after acoustic overexposure, leading to hyperactivity and thus tinnitus generation^18^. The studies outlined above illustrate that neurotransmission changes at varied structures (such as DCN, MGB, IC, auditory cortex) lead to an overall excitatory effect with decreased inhibitory effect, translating to hyperexcitability that is perceived as tinnitus by the patient.

#### 2.1.3 Maladaptive plasticity

Neural plasticity, in the simplest terms, is the ability of the nervous system to change and adapt via reorganization of the neurons in response to new stimuli^19^. This phenomenon has been extensively studied in relation to memory. Thus, a review relates the plasticity of memory and tinnitus. The authors state that a NMDA receptor’s 2B unit is involved during memory consolidation (a neural plasticity phenomenon) and similarly, 2B subunit antagonists in cochlear NMDA receptors eradicated long term noise-induced tinnitus in rats^20^. Clearly, NMDA receptor antagonism causing tinnitus eradication indicates neuronal plasticity is being altered at the onset of tinnitus. The term maladaptive plasticity involves “misdirected” learning and is indicated in other neurological pathologies such as neuropathic pain^21^. In the context of tinnitus, the ringing may initially be due to hearing loss damage at the level of the cochlea, but chronic tinnitus generation is sustained due to maladaptive changes in the auditory and non-auditory structures^21^. In later sections, maladaptive plasticity will be discussed at different levels of the auditory pathway and non-auditory structures, highlighting the key role this mechanism contributes to tinnitus generation and perception.

### 2.2 System level

#### 2.2.1 Auditory structures involved in tinnitus pathology

*2.2.1.1 Dorsal cochlear nucleus*

One of the first physiological hallmarks of tinnitus was cited at the level of the dorsal cochlear nucleus (DCN), terming it the tinnitus generator^8,22^. The main mechanism of DCN involvement includes increased neuronal synchrony^23-27^. For example, a rodent study demonstrated decreased synchronization of spontaneous firing due to blocked NMDA receptors in the fusiform cells of the DCN^23^. Furthermore, salicylate-induced tinnitus in guinea pigs demonstrated increased SFRs, synchrony and stimulus timing dependent plasticity^27^. The increased hyperactivity in the DCN seen as a consequence of decreased auditory nerve input is carried through to the inferior colliculus (IC), causing the IC to have elevated neuronal activity as well^24,28^. On the other hand, a comparison study on the DCN and IC reported hyperactivity in the IC to be lower than in the DCN and explores the possibility of independent mechanisms causing hyperactivity in each structure^29^.

Overall, as the previous section on the cellular level has established the link for hyperactivity and tinnitus, the increased neuronal activity in DCN only further supports that claim. The inter-relationship between DCN and IC needs to be investigated further to determine if preceding and/or succeeding structures in the auditory pathway impact the DCN/IC and consequently, contribute to tinnitus generation.

DCN studies have also explored the plasticity changes at this level as another cause for tinnitus. Two different studies performed on guinea pigs illustrated DCN’s fusiform cells experiencing plasticity changes^27^ and the other showed alterations in the DCN’s bimodal plasticity^5,30^.

DCN has a long-standing reputation as a tinnitus generator and, recent literature has only further proven its fundamental role. Therefore, the mechanism of DCN is largely undisputed and consistent.

*2.2.1.2 Inferior colliculus*

The inferior colliculus (IC) located in the midbrain of the brainstem is another auditory structure linked to tinnitus on the basis of hyperactivity^31,32^. A review cited multiple studies that all point to increased neural activity in the IC^31^, one of which included an animal study of salicylate-induced guinea pigs and rats demonstrating increased excitability. Another guinea pigs study demonstrated increased neural gap detection thresholds in the IC^33^. In contrast, one study demonstrated increased neuronal synchrony at the level of IC in guinea pigs post noise-induced trauma, but the hyperactivity was not significant between tinnitus and non-tinnitus guinea pig^34^.

In humans, one study demonstrated that the ablation of the DCN led to reduced IC hyperactivity, implicating not only IC’s role in tinnitus generation but also that its hyperexcitability is, to some extent, induced by DCN^35^. Contrarily, fMRI study done on IC and other structures did not demonstrate increased activity in tinnitus patients^36^. A similar sentiment is echoed by another study that states the activation of the IC was likely due to abnormal sound level tolerance rather than tinnitus^37^.

Although the animal studies make a strong case for IC to be considered for tinnitus pathology, the recent studies on tinnitus patient’s IC creates doubt. Therefore, the role of IC in tinnitus pathology is not as clear cut and requires greater investigation to verify its involvement.

*2.2.1.3 Medial geniculate body*

The medial geniculate body (MGB) of the thalamus is a higher center along the auditory pathway which integrates auditory and limbic information^38^. Like DCN and IC, MGB has also been implicated in tinnitus generation^38-40^although the current literature on it is limited. Animal models have demonstrated hyperactivity at the MGB^40^ but conversely, there is evidence to prove that there is reduced neuronal excitability instead^41^. Consequently, MGB’s role still requires more investigation to certify its significance in tinnitus generation.

On another note, the MGB projects to the amygdala which is a component of the limbic system involved in processing negative stimuli and emotions^42^. As discussed later on, tinnitus has an emotional component to its pathology therefore the link between the MGB and amygdala should be explored further to understand long-term tinnitus perception.

*2.2.1.4 Auditory cortex*

The auditory cortex (AC) is another well-established structure in tinnitus pathology as it is the final higher center at which auditory stimuli is processed. One study showed increasing intensity of perceived tinnitus with increased gamma band activity of the contralateral auditory cortex^43^ whilst another study demonstrated increased gamma band activity in the left and right primary and secondary auditory cortex of tinnitus patients^44^. An innovative functional near-infrared spectroscopy (fNIRS) approach demonstrated increased hemodynamic activity in the AC indicating plasticity changes^45^ whereas another fNIRS illustrated increased resting state functional activity in the AC of tinnitus patients vs. non tinnitus individuals^46^. A rat model illustrated augmented cortical excitability post- salicylate induced tinnitus^47^, again reiterating the cellular mechanism of hyperactivity^47^ discussed previously but now in the context of the AC. In conclusion, the AC has cemented its role in tinnitus pathology, likely through hyper-neuronal activity^47,48^, as it is the final center at which auditory stimuli are processed.

#### 2.2.2 Non-auditory structures involved in tinnitus pathology

*2.2.2.1 Parahippocampus*

The parahippocampal area plays a role in auditory habituation and since tinnitus perception is continuous (i.e., there is no habituation of the sound that is perceived), the role of the parahippocampus in perpetuating tinnitus and preventing its habituation becomes clear^49^. Recent literature supports its role in tinnitus, particularly, individuals with unilateral tinnitus showed increased high frequency activity in the right parahippocampal area with increased gamma band activity of contralateral parahippocampal area^49^. Another study recorded grey matter reduction in the parahippocampus^32^. The parahippocampus has also shown increased connections with non-auditory areas in chronic tinnitus^50^. Lastly, a meta-analysis mentions multiple fMRI’s that have been conducted, implicating the parahippocampal role in tinnitus pathology^51^. Overall, the parahippocampus' role is evidently significant in tinnitus perception.

*2.2.2.2 Dorsal anterior cingulate cortex*

The dorsal anterior cingulate cortex (dorsal ACC) has evidence to support its role in tinnitus distress networks. A blind source separation analysis on tinnitus networks and tinnitus distress compared resting state electrical activity of tinnitus patients with controls and compared low vs. high distress tinnitus patients^52^. The results of the study showed two anatomically specific networks (termed IC5 and IC6) with distress related differences in tinnitus patients when compared to the controls. Specifically, tinnitus distress created abnormal alpha and beta activity in the subgenual ACC extending to the pregenual and dorsal ACC and the ventromedial prefrontal cortex/orbitofrontal cortex, insula and parahippocampal area^52^. The implications of these results stresses upon the link between a tinnitus distress network playing a role in tinnitus pathology. Therefore, how important is the tinnitus distress network to tinnitus pathology itself? As of yet, more research in regard to the psychological aspect of tinnitus, in this case a distress network, needs to be conducted. The distress network is discussed in another section below.

*2.2.2.3 Ventral prefrontal cortex*

A 3-part MRI studies that used voxel-based morphometry identified a decrease in gray matter in the subcallosal regions (specifically in the ventral prefrontal cortex) in tinnitus patients compared to controls^53^. An article on frontostriatal gating cites multiple studies that have also shown gray matter reduction in this same region^54^. The ventromedial prefrontal cortex (vmPFC) determines the extent to which the abnormal auditory activity is perceived consciously as tinnitus. This function suggests that gray matter reduction here indicates that the tinnitus suppression mechanism that the vmPFC should carry out is hindered, and thus tinnitus perception can occur.

The frontostriatal system (includes vmPFC and nucleus accumbens) assigns subjective value to external or internal sensory signals^54^. In the case of tinnitus, changes in the input to this system can cause dysfunction in the valuation process and lead to a neutral stimulus becoming a negative stimulus and thus cause tinnitus perception^54^.

*2.2.2.4 Insula*

The insula, like the dorsal ACC, has been implicated in tinnitus distress^49,55^. Insula plays a role in the autonomic nervous system therefore, the tinnitus distress correlated with sympathetic activation proves the insula as a strong candidate for being involved^49^. Furthermore, the alpha activity in the left and right anterior insula was seen in patients with severe tinnitus distress^49^. The altered alpha activity seen in the insula as evidence for tinnitus pathology also strengthens the claims given in the paragraph above about altered alpha and gamma activity in the dorsal ACC.

A meta-analysis states that neuromodulation of the insula of tinnitus patients was the strongest compared to other regions (amygdala, parahippocampus, ACC), indicating that it is likely involved in auditory processing which is impaired in tinnitus^50^.

The insula is also considered in a salience network responsible for sensory integration^56^. This network becomes relevant in the context of tinnitus in which there is sensory processing of a sound that is not actually elicited by an auditory stimulus.

*2.2.2.5 Orbitofrontal cortex*

Like the insula, the orbitofrontal cortex (OFC) is involved in the emotional processing of sounds and is indicated in tinnitus distress networks^49,55^. These findings are further supported by a study that found tinnitus patients (especially females) were more emotionally responsive to tinnitus distress and had increased synchronized connectivity between the OFC and insula^49^. Therefore, the role of the OFC in tinnitus generation is indicated by the role it plays in the tinnitus distress network.

*2.2.2.6 Posterior cingulate cortex and precuneus*

There is the concept of a network termed the brain default network which consists of the following structures: parahippocampal area, posterior cingulate cortex and precuneus^49^. In the case of tinnitus patients, these 3 regions are more active during tinnitus perception.

#### 2.2.3 Functional connectivity in the higher centers

This section of the review covers how different functional connectivities in the higher centers causes tinnitus generation and tinnitus perception. It is important to note the distinction between generation and perception; the former is at the level of the auditory system and the latter occurs due to the abnormal coupling of higher order centers of the brain with regions outside the auditory system^57^.

*2.2.3.1 Alterations in RSFC*

With the shift towards understanding that non-auditory regions are involved in long term tinnitus perception, the concept of altered RSFC has been investigated. FMRI, EEG and MEG studies of the resting state in tinnitus patients have been studied but clarity in terms of which structures are involved still requires more research^58^. A specific study done on RSFC measured via fNIRS demonstrated increased hemodynamic activity in the auditory and selected adjacent non auditory cortices in tinnitus patients after sound stimulation and 5/9 non regions of interest (ROI) exhibited an increase in connectivity to the rest of the regions that were measured, suggesting that non-auditory regions contribute to chronic tinnitus perception^59^. An fMRI study compared the auditory resting state network (RSN) connectivity in tinnitus patients vs. healthy individuals. The study demonstrated that chronic tinnitus patients had increased connectivity in the brainstem, cerebellum, right basal ganglia, parahippocampal areas, right frontal and parietal areas, left sensorimotor areas and left superior temporal region^58^. Another fMRI study found an atypical RSN in tinnitus patients consisting of the medial Heschl’s gyrus (i.e., the AC), inferior colliculus, mediodorsal nucleus, striatum, OFC, and lateral prefrontal cortex^58^. Some of these structures (AC, IC, OFC) have been discussed in previous sections, providing credibility to the presence of this RSN in tinnitus.

*2.2.3.2 Auditory - limbic association*

A study using blood oxygenation level dependent response tests (BOLD) illustrated that functional connectivity of the brain was altered in bothersome tinnitus but not in non-bothersome tinnitus which indicates that the emotional and attentional aspect plays an important role in chronic tinnitus perception^60^. This relationship between auditory and limbic regions (which are the hubs for emotional and attentional processes) dates back to 1990, described by Jastreboff in his neurophysiological model of tinnitus^611^. A more recent article outlines the “noise cancellation” process of the limbic system which eliminates unwanted sound signals by sending it to the *inhibitory* thalamic reticular nucleus, which removes the sound signal from reaching the auditory cortex^62^. However, in tinnitus, this noise cancellation system is impaired leading to the sound signal reaching the AC and causing cortical reorganization at this level that translates to tinnitus perception^62^. Evidently, the limbic system plays a cardinal role in tinnitus pathology, especially chronic tinnitus generation in which the functional connectivities shift from the auditory system to a more diffused location throughout multiple auditory and non-auditory regions (i.e., limbic system) of the brain. On a different note, the limbic system is involved in tinnitus distress networks as well, since a MRI-based study confirmed that tinnitus patients suffer from psychological distress which is strongly associated with the limbic system^32^. Furthermore, the study cites another functional research that linked the parahippocampus (part of the limbic system) to distress^32^.

*2.2.3.3 Global tinnitus network*

Up until now, it has become clear that the initial onset of tinnitus affects the auditory system primarily (usually in the form of hyperactivity) however, chronic tinnitus generation mechanisms shift towards integrating non-auditory higher centers with changed functional connectivities.

Therefore, the development of a global tinnitus network has become prevalent in recent tinnitus research. This network consists of long-range cortical connections that are outside of the central auditory system. A particular study relates the global workspace model of Dehaene-Changeux to a global tinnitus network. Dehaene-Changeux’s model explains that what one experiences consciously is a consequence of selective amplification and global broadcasting of the specific piece of information to multiple distant areas^63^. The study attempts to probe this tinnitus network via sound stimuli resembling the tinnitus tone of the patient and then observing the effect on the functional connectivity of the network^9^. Eight regions were studied yielding the following results: anterior cingulate cortex (ACC) with right frontal and ACC with right parietal showed meaningful correlation indicating tinnitus intrusiveness (i.e., how bothersome the tinnitus is)^9^. These correlations were only found in tinnitus subjects and not in control conditions further supporting that these specific connectivities within the brain are likely related to a global tinnitus network. While their results provide some evidence of such a network existing, it is possible that a general salience network (consisting of anterior insula and dorsal ACC^64^) was activated instead by the perceived importance of the tinnitus sound. Thus, more studies are needed to evaluate if there is a clear distinction between the general salience network and a specific tinnitus network. If a specific tinnitus network does exist, then more investigation on the structures involved needs to be explored.

*2.2.3.4 Depression and distress networks*

One study aimed to separate depression and distress networks caused by tinnitus from the neural changes i.e. tinnitus intensity networks. A source analysis of resting state EEG activity was done which demonstrated a positive correlation between those who had a higher score for tinnitus-related distress (measured via the Tinnitus Questionnaire) and increased activity in the frontopolar, OFC, sgACC and pgACC^65^. There was also a positive correlation between increased BDI (Beck Depression Inventory-II) with the frontopolar, OFC, and sgACC regions^65^. As discussed before, ACC plays a role in the emotional processing network thus the positive correlation between tinnitus distress and ACC further supports this statement (see dorsal ACC section for its role in tinnitus distress networks). Further, the OFC is involved in the pathophysiology of depression and it is positively correlated in this study of tinnitus patients, again demonstrating the presence of a potential depression network in tinnitus^65^. The paper concludes that the parahippocampal area showed increased activity in those with higher BDI scores and this region is likely the link between a tinnitus network and the attention-emotion circuit related to tinnitus distress, underscoring this region’s role in tinnitus pathology.

### 2.3 Others

#### 2.3.1 Thalamocortical dysrhythmia (TCD)

TCD claims that in a state of deaffernation, the dominant alpha band activity in the thalamus reduces to theta band activity with gamma band activity surrounding the theta area^66^. The increased gamma band activity is due to reduced GABA_A_-mediated lateral inhibition^67^ while on a molecular level, the deinactivation of T-type Ca channels at the thalamic relay cells are involved^66^. This switch from alpha activity to theta and gamma activity is a feature of tinnitus thus implicating TCD mechanisms in tinnitus pathology^66,67^.

## 3. Treatment and Therapeutics

**Figure 2:**
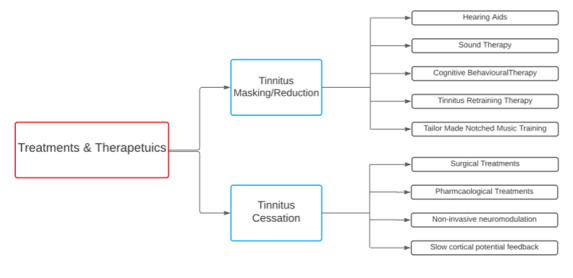
Treatments and therapeutics flowchart

Current treatments and therapeutics can be divided into two categories: tinnitus masking/ reduction and tinnitus cessation (Figure 2). Currently, tinnitus masking treatments play a greater role in the first line treatment of tinnitus as they are more effective and easier to implement for tinnitus patients, allowing the patient’s long-term outcome to be improved.

### 3.1 Tinnitus masking/reduction

#### 3.1.1 Hearing aids

Hearing aids are useful in tinnitus patients with or without hearing loss. In the case of the latter, the hearing aids can augment the peripheral noise as a means to mask the tinnitus sound and help the patient focus on the ambient noises rather than their tinnitus^2^. In the case of concurrent hearing loss and tinnitus, the hearing aids serve a dual purpose^2^.

#### 3.1.2 Sound therapy

This therapy involves sound production that stimulates the auditory system causing the patient to focus on that sound rather than their tinnitus thereby reducing their intensity and perception of tinnitus temporarily. This can be achieved through hearing aids or through any system that can produce sound (ex. musical player)^2^. There is evidence in support of sound therapy being a beneficial treatment for tinnitus^68-70^. One study positively correlates tinnitus relief with sound therapy^68^, while another study found that customized sound therapy (i.e., sound production catered to the individual’s tinnitus type) improved tinnitus loudness^69^. However, a meta-analysis on sound therapy concludes that there is no evidence to support the therapeutic effect of this therapy^71^. Currently, the use of sound therapy is criticized for not having sufficient evidence to prove its efficacy. Therefore, sound therapy is not a first line treatment for tinnitus and instead the preference of the patient is considered for choosing to avail this treatment option^71^. See TRT section below for its uses in that therapy.

#### 3.1.3 Cognitive behavioral therapy

Oftentimes, the onset of tinnitus leads to emotional disturbances such as anxiety and depression in the patient as they attempt to grapple with this new condition in their lives. Thus, CBT is beneficial in reducing the patient’s negative response to tinnitus via counseling and relaxation techniques aimed at their anxiety/depression^72^. Notably, a recent systematic review on CBT illustrates that although CBT is more beneficial than no treatment at all for tinnitus management, it is still not highly efficacious^73^. The review compared CBT to other therapies (e.g., tinnitus retraining therapy), establishing that it can relieve some depression symptoms and reduce tinnitus impact on quality of life but there is no evidence of this 6-12 months post-treatment^73^.

Although CBT may not hold extreme efficacy on its own, a recent study showed promising results by combining CBT with music therapy^73,74^. The CBT-Music group showed significant improvement in their tinnitus perception relative to the CBT and Music groups^73,74^.

In conclusion, implementation of CBT is not harmful for tinnitus patients, but its efficacy and benefits are minimal.

#### 3.1.4 Tinnitus retraining therapy (sound therapy type)

TRT was described by Jastreboff’s neuro-physiological model back in the 1990s^75^. The therapy itself is twofold: counselling is given to convert tinnitus stimuli into a neutral stimuli and sound therapy is provided to reduce hyperactivity of the tinnitus related neurons overall aiming to habituate one to their tinnitus^76^. There is evidence to support the efficacy of this therapy^76-78^ and with continued treatment, TRT may prove to be one of the best treatment options for tinnitus^76^.

#### 3.1.5 Tailor made notched music training (TMNMT)

It is an acoustic neuromodulation method in which the notched music excludes one octave width of the frequency band centered at the individual's tinnitus frequency range (i.e., the notched range)^7,79^. Therefore, the frequency in the tinnitus range is not stimulated but the neurons situated adjacent to this area are stimulated and due to lateral inhibition, they exert an inhibitory influence on the neurons in the ‘notched’ range. A double-blind study demonstrated significant decreases in tinnitus loudness in the tinnitus group^80^. Furthermore, MEG results showed decreased synchrony of neurons in cortical areas contributing to tinnitus perception after using TMNMT^80^.

### 3.2 Tinnitus cessation

#### 3.2.1 Surgical treatments

*3.2.1.1 Cochlear implants*

Surgical placement of cochlear implants is a beneficial treatment in individuals with concurrent sensorineural loss and tinnitus^81,82.^ A longitudinal prospective study done on 142 cochlear implant patients assessed their tinnitus handicap inventory (THI) before and after implantation^82^. The results were statistically significant indicating that the implants had a suppressive effect on tinnitus thereby reducing tinnitus perception. In 37% of the individuals, there was complete tinnitus suppression^82^, validating it as a potential tinnitus cessation treatment.

*3.2.1.2 Deep brain stimulation*

A Phase 1 trial conducted deep brain stimulation (DBS) of the caudate nucleus in treatment-resistance tinnitus patients. The study showed promising results in which patients indicated lower Tinnitus Functional Index (TFI) and tinnitus handicap inventory scores post-DBS. Currently, this surgical neuromodulation is one of the newest treatments present for tinnitus cessation^83^.

DBS has been used at other targets for tinnitus cessation such as the subthalamic nucleus, globus pallidus internus and ventral intermediate nucleus of the thalamus. The study itself demonstrated that the subthalamic nucleus proved to be the most beneficial target for tinnitus^84^.

Overall, more research is required to test the feasibility of DBS treatment’s long-term efficacy and which DBS targets in the brain are most effective in eliminating tinnitus.

*3.2.1.3 Microvascular decompression*

A review on surgical treatments for tinnitus covers microvascular decompression of the cochlear nerve to relieve tinnitus in patients that were experiencing chronic compression of this nerve. The review cites a study that reported the improvement of tinnitus in 7 out of 13 patients with improved scores on the TQ (Tinnitus Questionnaire)^81^.

#### 3.2.2 Pharmacological treatments

Currently, there are no pharmacological treatments available for tinnitus loudness or distress^85^. This review aimed to look at treatments in the past five years and unfortunately no sustainable treatment has been found as of yet for tinnitus. One promising treatment from 2015 indicates NMDA receptor antagonism in rat cochlea led to tinnitus reduction^86^ however, further trials have yet to be conducted.

Despite the presence of tinnitus distress networks and the causal link between tinnitus and depression, current guidelines advise against antidepressant, anticonvulsant or anxiolytic medication for patients with bothersome tinnitus^72^. However, patients with pre-existing anxiety and depression can use antidepressants/selective serotonin reuptake inhibitors to manage tinnitus symptoms^72^.

#### 3.2.3 Non-invasive neuromodulation

*3.2.3.1 Transcranial direct current stimulation (tDCS)*

Mechanism behind tDCS method constitutes modulation of cortical excitability via anodal and cathodal stimulation in which the cathodal tDCS is usually placed over the auditory cortex to reduce tinnitus related hyperactivity^7,79^. A double-blind placebo-controlled crossover design demonstrated a beneficial short-term effect in tinnitus intensity for 7 out of 20 patients when tDCS was applied over the left temporoparietal area^87^.

Contrarily, a bifrontal tDSC therapy in distressed patients with *severe* tinnitus caused alleviation of tinnitus related distress but treatment did not correlate with reduction in tinnitus loudness^88^. Furthermore, a combined therapy of tDSC over auditory cortex with tailor made notched music training in tonal tinnitus with concurrent severe hearing loss indicates there was reduction in tinnitus related distress^7,79^.

Majority of the benefit of tDCS has been in the form of reducing tinnitus distress rather than reducing tinnitus loudness itself. Regardless, the treatment still needs more investigation to establish general treatment protocol.

*3.2.3.2 Repetitive transcranial magnetic stimulation (rTMS)*

RTMS has been a controversial treatment option due to conflicting results, evidence of high placebo effect, and variability in the treatment’s effectiveness and longevity^89^.

In earlier repetitive transcranial magnetic stimulation (rTMS) studies, the focus was on stimulating auditory cortices but recently, the focus has shifted to stimulation of non-auditory cortices^90^ with the advent of tinnitus networks involving non auditory structures. A blinded randomized control study demonstrated improved tinnitus handicap inventory and VAS scores (visual analogue scores) after dual-rTMS of the frontal and auditory cortex^90^. Furthermore, a systematic review confirmed the efficacy of rTMS for chronic tinnitus, citing that rTMS treatment showed efficacy at the one-week mark and continued to do so 6 months post-treatment^91^. On the contrary, another systematic review shows conflicting results, indicating that rTMS had little benefit in reducing the psychological issues of the patient, since there was no change in Tinnitus Questionnaires (TQ) and BDI (Beck's depression inventory)^92^. Overall, there is still contention over using tDCS or rTMS as a first line of treatment and more research is required to establish streamlined guidelines on treatment protocol that will prove to be effective long term for the patient.

*3.2.3.3 Slow cortical potential neurofeedback*

A case report of a 50-year-old male with chronic tinnitus underwent this treatment involving slow cortical potentials (regulate excitation thresholds that might be impaired in pathological conditions such as tinnitus)^93^. The patient reported a decrease in tinnitus loudness and pitch and these findings were supported by an EEG analysis showing close to normal changes in resting state activity of cortical areas implicated in tinnitus generation^93^. However, this case report is not substantial in indicating the efficacy of the treatment for the general population of tinnitus patients thus, more research with this treatment is required to establish its role as a viable treatment option.

## 4. Conclusion

Overall, tinnitus pathology is multifaceted as the disease is heterogeneous in nature. Thus, the mechanisms highlighted in the cellular level section should be considered together, in a stepwise fashion, for an in-depth understanding. The initial onset of tinnitus is considered at the cochlea, due to cochlear nerve deafferentation, leading to decreased auditory input to the auditory system. Consequently, the auditory system (and non-auditory structures) attempt to compensate leading to neurotransmission changes that result in hyperactivity. As discussed in the system-level section, hyperactivity is seen in auditory and non-auditory structures and is implicated in potential tinnitus networks which demonstrate a change in functional connectivities. The long-term consequence of these neuronal changes leads to more permanent neuronal plasticity changes in the auditory/non-auditory structures, leading to chronic tinnitus generation and perception.

Regarding treatments, no current tinnitus cessation treatments can guarantee tinnitus eradication. Pharmacological treatments have had a long history in tinnitus treatment research however, even currently, no such drug has prevailed to show benefit in tinnitus reduction or cessation. Consequently, tinnitus masking/reducing treatments *relatively* show better efficacy (e.g., TRT, TMNMT).

Some promising results in efficacy of DBS, tDCS and TMS treatments are met with issues regarding the absence of a standard treatment protocol nor having long-term efficacy. In conclusion, the shift towards understanding changes in functional connectivity and tinnitus networks has allowed treatment research to also shift towards targeting structures in the brain to reduce tinnitus perception. Although the current treatments are still being developed and refined, they still hold potential in eliminating tinnitus altogether.

## KEY POINTS

◊ *Tinnitus is a heterogeneous condition, considered as a symptom that is almost always associated with an underlying disease. *

**◊** *Investigations regarding a global tinnitus network, tinnitus-distress and depression networks are relatively newer in tinnitus research. Therefore, this domain of research requires further exploration to develop solid evidence of these network’s existence in tinnitus pathology.*

**◊** *Neuromodulation treatments, such as tDCS, rTMS and DBS, are being tested and show some promising results. However, further testing and trials are required to establish a standardized protocol and long term efficacy of the treatment.*

## References

[1] (2014). Tinnitus. NIH, Bethesda, MD, USA: NIDCD (National Institute on Deafness and Other Communication Disorders).

[2] Esmaili Aaron A, Renton John (2018). A review of tinnitus. Australian Journal of General Practice.

[3] Baguley David, McFerran Don, Hall Deborah (2013). Tinnitus. The Lancet.

[4] Han Byung In, Lee Ho Won, Kim Tae You, Lim Jun Seong, Shin Kyoung Sik (2009). Tinnitus: Characteristics, Causes, Mechanisms, and Treatments. Journal of Clinical Neurology.

[5] Koehler S. D., Shore S. E. (2013). Stimulus Timing-Dependent Plasticity in Dorsal Cochlear Nucleus Is Altered in Tinnitus. Journal of Neuroscience.

[6] Savage Julian, Cook Stephanie, Waddell Angus (2009). Tinnitus.. BMJ clinical evidence.

[7] Teismann Henning, Wollbrink Andreas, Okamoto Hidehiko, Schlaug Gottfried, Rudack Claudia, Pantev Christo (2014). Combining Transcranial Direct Current Stimulation and Tailor-Made Notched Music Training to Decrease Tinnitus-Related Distress – A Pilot Study. PLoS ONE.

[8] Shore Susan E., Roberts Larry E., Langguth Berthold (2016). Maladaptive plasticity in tinnitus — triggers, mechanisms and treatment. Nature Reviews Neurology.

[9] Schlee Winfried, Weisz Nathan, Bertrand Olivier, Hartmann Thomas, Elbert Thomas (2008). Using Auditory Steady State Responses to Outline the Functional Connectivity in the Tinnitus Brain. PLoS ONE.

[10] Takeuchi Naoyuki, Izumi Shin-Ichi (2012). Maladaptive Plasticity for Motor Recovery after Stroke: Mechanisms and Approaches. Neural Plasticity.

[11] Auerbach Benjamin D., Rodrigues Paulo V., Salvi Richard J. (2014). Central Gain Control in Tinnitus and Hyperacusis. Frontiers in Neurology.

[12] Baguley David, Andersson Gerhard, McFerran Don, McKenna Laurence (2013). Mechanisms of Tinnitus. Tinnitus: A Multidisciplinary Approach.

[13] Lee Ho Yun, Choi Myoung Su, Chang Dong Sik, Cho Chin-Saeng (2017). Combined Bifrontal Transcranial Direct Current Stimulation and Tailor-Made Notched Music Training in Chronic Tinnitus. Journal of Audiology and Otology.

[14] Richardson Ben D., Brozoski Thomas J., Ling Lynne L., Caspary Donald M. (2012). Targeting inhibitory neurotransmission in tinnitus. Brain Research.

[15] Pilati Nadia, Large Charles, Forsythe Ian D., Hamann Martine (2012). Acoustic over-exposure triggers burst firing in dorsal cochlear nucleus fusiform cells. Hearing Research.

[16] Yang S., Weiner B. D., Zhang L. S., Cho S.-J., Bao S. (2011). Homeostatic plasticity drives tinnitus perception in an animal model. Proceedings of the National Academy of Sciences.

[17] Brozoski Thomas J., Caspary Donald M., Bauer Carol A., Richardson Benjamin D. (2010). The effect of supplemental dietary Taurine on Tinnitus and auditory discrimination in an animal model. Hearing Research.

[18] Berger Joel I., Coomber Ben (2015). Tinnitus-Related Changes in the Inferior Colliculus. Frontiers in Neurology.

[19] (2020). Neuroplasticity. Physiopedia.

[20] Guitton Matthieu J. (2012). Tinnitus: pathology of synaptic plasticity at the cellular and system levels. Frontiers in Systems Neuroscience.

[21] Møller Aage R. (2016). Sensorineural Tinnitus: Its Pathology and Probable Therapies. International Journal of Otolaryngology.

[22] van Zwieten Gusta, Jahanshahi Ali, van Erp Marlieke L., Temel Yasin, Stokroos Robert J., Janssen Marcus L. F., Smit Jasper V. (2019). Alleviation of Tinnitus With High-Frequency Stimulation of the Dorsal Cochlear Nucleus: A Rodent Study. Trends in Hearing.

[23] Stefanescu Roxana A., Shore Susan E. (2015). NMDA Receptors Mediate Stimulus-Timing-Dependent Plasticity and Neural Synchrony in the Dorsal Cochlear Nucleus. Frontiers in Neural Circuits.

[24] Wu Calvin, Martel David T., Shore Susan E. (2016). Increased Synchrony and Bursting of Dorsal Cochlear Nucleus Fusiform Cells Correlate with Tinnitus. The Journal of Neuroscience.

[25] Dehmel S., Pradhan S., Koehler S., Bledsoe S., Shore S. (2012). Noise Overexposure Alters Long-Term Somatosensory-Auditory Processing in the Dorsal Cochlear Nucleus--Possible Basis for Tinnitus-Related Hyperactivity?. Journal of Neuroscience.

[26] Baizer Joan S., Manohar Senthilvelan, Paolone Nicholas A., Weinstock Nadav, Salvi Richard J. (2012). Understanding tinnitus: The dorsal cochlear nucleus, organization and plasticity. Brain Research.

[27] Martel David T., Pardo-Garcia Thibaut R., Shore Susan E. (2019). Dorsal Cochlear Nucleus Fusiform-cell Plasticity is Altered in Salicylate-induced Tinnitus. Neuroscience.

[28] Olsen Timothy, Capurro Alberto, Pilati Nadia, Large Charles H., Hamann Martine (2018). Kv3 K+ currents contribute to spike-timing in dorsal cochlear nucleus principal cells. Neuropharmacology.

[29] Manzoor N.F., Gao Y., Licari F., Kaltenbach J.A. (2013). Comparison and contrast of noise-induced hyperactivity in the dorsal cochlear nucleus and inferior colliculus. Hearing Research.

[30] Wu Calvin, Martel David T., Shore Susan E. (2015). Transcutaneous induction of stimulus-timing-dependent plasticity in dorsal cochlear nucleus. Frontiers in Systems Neuroscience.

[31] Palmer Alan R., Berger Joel I. (2018). Changes in the Inferior Colliculus Associated with Hearing Loss. The Oxford Handbook of the Auditory Brainstem.

[32] Besteher Bianca, Gaser Christian, Ivanšić Daniela, Guntinas-Lichius Orlando, Dobel Christian, Nenadić Igor (2019). Chronic tinnitus and the limbic system: Reappraising brain structural effects of distress and affective symptoms. NeuroImage: Clinical.

[33] Berger Joel I., Coomber Ben, Wells Tobias T., Wallace Mark N., Palmer Alan R. (2014). Changes in the Response Properties of Inferior Colliculus Neurons Relating to Tinnitus. Frontiers in Neurology.

[34] Coomber Ben, Berger Joel I., Kowalkowski Victoria L., Shackleton Trevor M., Palmer Alan R., Wallace Mark N. (2014). Neural changes accompanying tinnitus following unilateral acoustic trauma in the guinea pig. European Journal of Neuroscience.

[35] Manzoor N. F., Licari F. G., Klapchar M., Elkin R. L., Gao Y., Chen G., Kaltenbach J. A. (2012). Noise-induced hyperactivity in the inferior colliculus: its relationship with hyperactivity in the dorsal cochlear nucleus. Journal of Neurophysiology.

[36] Lanting Cornelis P., de Kleine Emile, Langers Dave R. M., van Dijk Pim (2014). Unilateral Tinnitus: Changes in Connectivity and Response Lateralization Measured with fMRI. PLoS ONE.

[37] Boyen Kris, de Kleine Emile, van Dijk Pim, Langers Dave R.M. (2014). Tinnitus-related dissociation between cortical and subcortical neural activity in humans with mild to moderate sensorineural hearing loss. Hearing Research.

[38] Xia Chenchen, Yin Manli, Wu Cong, Ji Yonghua, Zhou You (2020). Neuroglial activation in the auditory cortex and medial geniculate body of salicylate-induced tinnitus rats.. American journal of translational research.

[39] van Zwieten Gusta, Janssen Marcus L. F., Smit Jasper V., Janssen A. Miranda L., Roet Milaine, Jahanshahi Ali, Stokroos Robert J., Temel Yasin (2018). Inhibition of Experimental Tinnitus With High Frequency Stimulation of the Rat Medial Geniculate Body. Neuromodulation: Technology at the Neural Interface.

[40] Kalappa Bopanna I., Brozoski Thomas J., Turner Jeremy G., Caspary Donald M. (2014). Single unit hyperactivity and bursting in the auditory thalamus of awake rats directly correlates with behavioural evidence of tinnitus. The Journal of Physiology.

[41] Su Yan-Yan, Luo Bin, Jin Yan, Wu Shu-Hui, Lobarinas Edward, Salvi Richard J., Chen Lin (2012). Altered Neuronal Intrinsic Properties and Reduced Synaptic Transmission of the Rat's Medial Geniculate Body in Salicylate-Induced Tinnitus. PLoS ONE.

[42] Caspary Donald M., Llano Daniel A. (2017). Auditory thalamic circuits and GABAA receptor function: Putative mechanisms in tinnitus pathology. Hearing Research.

[43] van der Loo Elsa, Gais Steffen, Congedo Marco, Vanneste Sven, Plazier Mark, Menovsky Tomas, Van de Heyning Paul, De Ridder Dirk (2009). Tinnitus Intensity Dependent Gamma Oscillations of the Contralateral Auditory Cortex. PLoS ONE.

[44] Vanneste S., Van de Heyning P., De Ridder D. (2011). Contralateral parahippocampal gamma-band activity determines noise-like tinnitus laterality: a region of interest analysis. Neuroscience.

[45] Zhai Tianqu, Ash‐Rafzadeh Angela, Hu Xiaosu, Kim Jessica, San Juan Juan D., Filipiak Charles, Guo Kaiwen, Islam Mohammed N., Kovelman Ioulia, Basura Gregory J. (2020). Tinnitus and auditory cortex; Using adapted functional
near‐infrared‐spectroscopy
to expand brain imaging in humans. Laryngoscope Investigative Otolaryngology.

[46] San Juan Juan D., Zhai Tianqu, Ash-Rafzadeh Angela, Hu Xiao-Su, Kim Jessica, Filipak Charles, Guo Kaiwen, Islam Mohammed N., Kovelman Ioulia, Basura Gregory J. (2020). Tinnitus and auditory cortex: using adapted functional near-infrared spectroscopy to measure resting-state functional connectivity. NeuroReport.

[47] Yi Bin, Hu Shousen, Zuo Chuantao, Jiao Fangyang, Lv Jingrong, Chen Dongye, Ma Yufei, Chen Jianyong, Mei Ling, Wang Xueling, Huang Zhiwu, Wu Hao (2016). Effects of long-term salicylate administration on synaptic ultrastructure and metabolic activity in the rat CNS. Scientific Reports.

[48] Wu Cong, Wu Xu, Yi Bin, Cui Mengchen, Wang Xueling, Wang Qixuan, Wu Hao, Huang Zhiwu (2018). Changes in GABA and glutamate receptors on auditory cortical excitatory neurons in a rat model of salicylate-induced tinnitus.. American journal of translational research.

[49] Vanneste Sven, De Ridder Dirk (2012). The auditory and non-auditory brain areas involved in tinnitus. An emergent property of multiple parallel overlapping subnetworks. Frontiers in Systems Neuroscience.

[50] Lefebvre-Demers Mathilde, Doyon Nicolas, Fecteau Shirley (2021). Non-invasive neuromodulation for tinnitus: A meta-analysis and modeling studies. Brain Stimulation.

[51] Chen Yu-Chen, Wang Fang, Wang Jie, Bo Fan, Xia Wenqing, Gu Jian-Ping, Yin Xindao (2017). Resting-State Brain Abnormalities in Chronic Subjective Tinnitus: A Meta-Analysis. Frontiers in Human Neuroscience.

[52] De Ridder Dirk, Vanneste Sven, Congedo Marco (2011). The Distressed Brain: A Group Blind Source Separation Analysis on Tinnitus. PLoS ONE.

[53] Seydell-Greenwald Anna, Leaver Amber M., Turesky Ted K., Morgan Susan, Kim Hung J., Rauschecker Josef P. (2012). Functional MRI evidence for a role of ventral prefrontal cortex in tinnitus. Brain Research.

[54] Rauschecker Josef P., May Elisabeth S., Maudoux Audrey, Ploner Markus (2015). Frontostriatal Gating of Tinnitus and Chronic Pain. Trends in Cognitive Sciences.

[55] Golm Dennis, Schmidt-Samoa Carsten, Dechent Peter, Kröner-Herwig Birgit (2016). Tinnitus- related distress: evidence from fMRI of an emotional stroop task. BMC Ear, Nose and Throat Disorders.

[56] Xu Xiao-Min, Jiao Yun, Tang Tian-Yu, Lu Chun-Qiang, Zhang Jian, Salvi Richard, Teng Gao-Jun (2019). Altered Spatial and Temporal Brain Connectivity in the Salience Network of Sensorineural Hearing Loss and Tinnitus. Frontiers in Neuroscience.

[57] Schlee Winfried, Hartmann Thomas, Langguth Berthold, Weisz Nathan (2009). Abnormal resting-state cortical coupling in chronic tinnitus. BMC Neuroscience.

[58] Leaver Amber M., Turesky Ted K., Seydell-Greenwald Anna, Morgan Susan, Kim Hung J., Rauschecker Josef P. (2016). Intrinsic network activity in tinnitus investigated using functional MRI. Human Brain Mapping.

[59] San Juan Juan, Hu Xiao-Su, Issa Mohamad, Bisconti Silvia, Kovelman Ioulia, Kileny Paul, Basura Gregory (2017). Tinnitus alters resting state functional connectivity (RSFC) in human auditory and non-auditory brain regions as measured by functional near-infrared spectroscopy (fNIRS). PLOS ONE.

[60] Burton Harold, Wineland Andre, Bhattacharya Mousumi, Nicklaus Joyce, Garcia Keith S, Piccirillo Jay F (2012). Altered networks in bothersome tinnitus: a functional connectivity study. BMC Neuroscience.

[61] Husain Fatima T., Schmidt Sara A. (2014). Using resting state functional connectivity to unravel networks of tinnitus. Hearing Research.

[62] Rauschecker Josef P., Leaver Amber M., Mühlau Mark (2010). Tuning Out the Noise: Limbic-Auditory Interactions in Tinnitus. Neuron.

[63] Dehaene S., Kerszberg M., Changeux J.-P. (1998). A neuronal model of a global workspace in effortful cognitive tasks. Proceedings of the National Academy of Sciences.

[64] (2020). Encyclopedia of Computational Neuroscience.

[65] Joos Kathleen, Vanneste Sven, De Ridder Dirk (2012). Disentangling Depression and Distress Networks in the Tinnitus Brain. PLoS ONE.

[66] De Ridder Dirk, Vanneste Sven, Langguth Berthold, Llinas Rodolfo (2015). Thalamocortical Dysrhythmia: A Theoretical Update in Tinnitus. Frontiers in Neurology.

[67] Lanting C.P., De Kleine E., Bartels H., Van Dijk P. (2008). Functional imaging of unilateral tinnitus using fMRI. Acta Oto-Laryngologica.

[68] Han Lv, Pengfei Zhao, Chunli Liu, Zhaodi Wang, Xindi Wang, Qian Chen, Shusheng Gong, Zhenchang Wang (2020). The effects of sound therapy in tinnitus are characterized by altered limbic and auditory networks.. Brain communications.

[69] Mahboubi Hossein, Haidar Yarah M., Kiumehr Saman, Ziai Kasra, Djalilian Hamid R. (2017). Customized Versus Noncustomized Sound Therapy for Treatment of Tinnitus: A Randomized Crossover Clinical Trial. Annals of Otology, Rhinology & Laryngology.

[70] Chen Qian, Lv Han, Wang Zhaodi, Wei Xuan, Zhao Pengfei, Yang Zhenghan, Gong Shusheng, Wang Zhenchang (2021). Brain Structural and Functional Reorganization in Tinnitus Patients Without Hearing Loss After Sound Therapy: A Preliminary Longitudinal Study. Frontiers in Neuroscience.

[71] Sereda Magdalena, Xia Jun, El Refaie Amr, Hall Deborah A, Hoare Derek J (2018). Sound therapy (using amplification devices and/or sound generators) for tinnitus. Cochrane Database of Systematic Reviews.

[72] Wu Vincent, Cooke Bonnie, Eitutis Susan, Simpson Matthew T W, Beyea Jason A (2018). Approach to tinnitus management.. Canadian family physician Medecin de famille canadien.

[73] Feng Tianci, Wang Mingxia, Xiong Hao, Zheng Yiqing, Yang Haidi (2020). Efficacy of an Integrative Treatment for Tinnitus Combining Music and Cognitive-Behavioral Therapy—Assessed With Behavioral and EEG Data. Frontiers in Integrative Neuroscience.

[74] Ugwuanyi Christian S, Ede Moses O, Onyishi Charity N, Ossai Osita V, Nwokenna Edith N, Obikwelu Lauretta C, Ikechukwu-Ilomuanya Amaka, Amoke Chijioke V, Okeke Agnes O, Ene Catherine U, Offordile Edmund E, Ozoemena Lilian C, Nweke Maduka L (2020). Effect of cognitive-behavioral therapy with music therapy in reducing physics test anxiety among students as measured by generalized test anxiety scale.. Medicine.

[75] Phillips John S, McFerran Don (2019). Neurophysiological model-based treatments for tinnitus. Cochrane Database of Systematic Reviews.

[76] Cuesta María, Cobo Pedro (2020). Broadband Sound Equalized by The Hearing Loss Curves as an Improved Stimulus for Tinnitus Retraining Therapy-A Pilot, Non-Controlled Observational Study. The Journal of International Advanced Otology.

[77] Nemade Sanjana Vijay, Shinde Kiran Jaywant (2019). Clinical Efficacy of Tinnitus Retraining Therapy Based on Tinnitus Questionnaire Score and Visual Analogue Scale Score in Patients with Subjective Tinnitus. Turkish Archives of Otorhinolaryngology.

[78] Reddy K. Vasu Kumar, Chaitanya V. Krishna, Babu G. Ramesh (2018). Efficacy of Tinnitus Retraining Therapy, A Modish Management of Tinnitus: Our Experience. Indian Journal of Otolaryngology and Head & Neck Surgery.

[79] Teismann Henning, Okamoto Hidehiko, Pantev Christo (2011). Short and Intense Tailor-Made Notched Music Training against Tinnitus: The Tinnitus Frequency Matters. PLoS ONE.

[80] Okamoto H., Stracke H., Stoll W., Pantev C. (2009). Listening to tailor-made notched music reduces tinnitus loudness and tinnitus-related auditory cortex activity. Proceedings of the National Academy of Sciences.

[81] De Salles AntonioA.F., Soleymani Teo, Pezeshkian Patrick, Gorgulho AlessandraA, Pieton David, Miller Patrick, Pouratian Nader (2011). Surgical approaches to tinnitus treatment: A review and novel approaches. Surgical Neurology International.

[82] Amoodi Hosam A., Mick Paul T., Shipp David B., Friesen Lendra M., Nedzelski Julian M., Chen Joseph M., Lin Vincent Y. W. (2011). The effects of unilateral cochlear implantation on the tinnitus handicap inventory and the influence on quality of life. The Laryngoscope.

[83] Cheung Steven W., Racine Caroline A., Henderson-Sabes Jennifer, Demopoulos Carly, Molinaro Annette M., Heath Susan, Nagarajan Srikantan S., Bourne Andrea L., Rietcheck John E., Wang Sarah S., Larson Paul S. (2020). Phase I trial of caudate deep brain stimulation for treatment-resistant tinnitus. Journal of Neurosurgery.

[84] Smit JasperV, Janssen MarcusL. F., Engelhard Malou, de Bie RobM. A., Schuurman PRichard, Contarino MariaF, Mosch Arne, Temel Yasin, Stokroos RobertJ (2016). The impact of deep brain stimulation on tinnitus. Surgical Neurology International.

[85] Kleinjung Tobias, Langguth Berthold (2020). Avenue for Future Tinnitus Treatments. Otolaryngologic Clinics of North America.

[86] Bing Dan, Lee Sze Chim, Campanelli Dario, Xiong Hao, Matsumoto Masahiro, Panford-Walsh Rama, Wolpert Stephan, Praetorius Mark, Zimmermann Ulrike, Chu Hanqi, Knipper Marlies, Rüttiger Lukas, Singer Wibke (2015). Cochlear NMDA Receptors as a Therapeutic Target of Noise-Induced Tinnitus. Cellular Physiology and Biochemistry.

[87] Garin Pierre, Gilain Chantal, Damme Jean-Philippe, Fays Katalin, Jamart Jacques, Ossemann Michel, Vandermeeren Yves (2011). Short- and long-lasting tinnitus relief induced by transcranial direct current stimulation. Journal of Neurology.

[88] Lee Ho Yun (2019). Adjunctive Role of Bifrontal Transcranial Direct Current Stimulation in Distressed Patients with Severe Tinnitus. Journal of Korean Medical Science.

[89] Londero A., Bonfils P., Lefaucheur J.P. (2018). Transcranial magnetic stimulation and subjective tinnitus. A review of the literature, 2014–2016. European Annals of Otorhinolaryngology, Head and Neck Diseases.

[90] Noh Tae-Soo, Kyong Jeong-Sug, Park Moo Kyun, Lee Jun Ho, Oh Seung Ha, Suh Myung-Whan (2020). Dual-site rTMS is More Effective than Single-site
rTMS in Tinnitus Patients: A Blinded Randomized Controlled Trial. Brain Topography.

[91] Liang Zhengrong, Yang Haidi, Cheng Gui, Huang Lingfei, Zhang Tao, Jia Haiying (2020). Repetitive Transcranial Magnetic Stimulation on Chronic Tinnitus: A Systematic Review and Meta-Analysis.

[92] Yin Lu, Chen Xiao, Lu Xingang, An Yun, Zhang Tao, Yan Juntao (2021). An updated meta-analysis: repetitive transcranial magnetic stimulation for treating tinnitus. Journal of International Medical Research.

[93] Milner Rafał, Lewandowska Monika, Ganc Małgorzata, Cieśla Katarzyna, Niedziałek Iwona, Skarżyński Henryk (2015). Slow Cortical Potential Neurofeedback in Chronic Tinnitus Therapy: A Case Report. Applied Psychophysiology and Biofeedback.

